# Production of polyhydroxyalkanoates (PHAs) by *Bacillus megaterium* using food waste acidogenic fermentation-derived volatile fatty acids

**DOI:** 10.1080/21655979.2021.1935524

**Published:** 2021-06-11

**Authors:** Danh H. Vu, Steven Wainaina, Mohammad J. Taherzadeh, Dan Åkesson, Jorge A. Ferreira

**Affiliations:** Swedish Centre for Resource Recovery, University of Borås, Sweden

**Keywords:** Acidogenic fermentation, *bacillus megaterium*, biopolymers, food waste, polyhydroxyalkanoates, polyhydroxybutyrate, volatile fatty acids

## Abstract

High production costs still hamper fast expansion of commercial production of polyhydroxyalkanoates (PHAs). This problem is greatly related to the cultivation medium which accounts for up to 50% of the whole process costs. The aim of this research work was to evaluate the potential of using volatile fatty acids (VFAs), derived from acidogenic fermentation of food waste, as inexpensive carbon sources for the production of PHAs through bacterial cultivation. *Bacillus megaterium* could assimilate glucose, acetic acid, butyric acid, and caproic acid as single carbon sources in synthetic medium with maximum PHAs production yields of 9–11%, on a cell dry weight basis. Single carbon sources were then replaced by a mixture of synthetic VFAs and by a VFAs-rich stream from the acidogenic fermentation of food waste. After 72 h of cultivation, the VFAs were almost fully consumed by the bacterium in both media and PHAs production yields of 9–10%, on cell dry weight basis, were obtained. The usage of VFAs mixture was found to be beneficial for the bacterial growth that tackled the inhibition of propionic acid, iso-butyric acid, and valeric acid when these volatile fatty acids were used as single carbon sources. The extracted PHAs were revealed to be polyhydroxybutyrate (PHB) by characterization methods of Fourier-transform infrared spectroscopy (FTIR) and differential scanning calorimetry (DSC). The obtained results proved the possibility of using VFAs from acidogenic fermentation of food waste as a cheap substrate to reduce the cost of PHAs production.

## GRAPHICAL ABSTRACT

**Figure:**
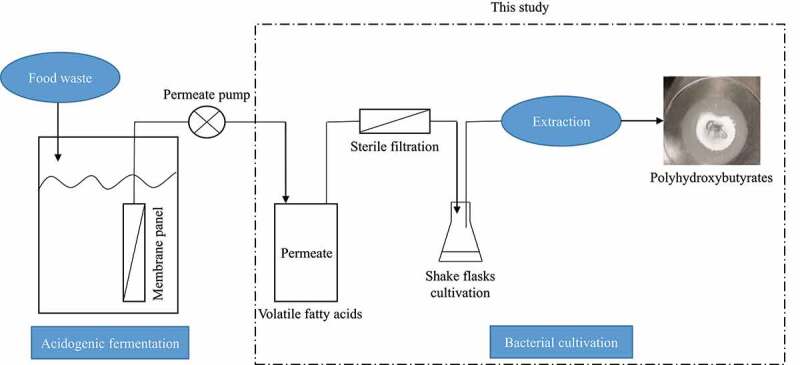

## 1. Introduction

The dependence of the modern society on petroleum-based plastics is undeniable due to its great performance in terms of physical properties and durability [1]. This, in turn, has been leading to one of the most severe environmental crises, namely plastic pollution. The detrimental impact of this problem on the ecosystem has been revealed by several scientific studies pointing out the urgent need of developing more environmentally friendly materials [2]. In this context, the ideal solution is to use bioplastics or biopolymers derived from renewable biomass resources or produced by a variety of microorganisms [3]. Several products have reached the market including thermoplastic starch, bio-polyamides, bio-polyols, cellulose-based polymers, polylactic acid (PLA), and polyhydroxyalkanoates (PHAs) [4]. Among these, only PLA and PHAs possess similar thermal and mechanical properties to those of conventional plastics [4].

Polyhydroxyalkanoates (PHAs) are a group of natural polyesters accumulated as intracellular granules by a variety of microorganisms which production is generally triggered by limiting nutrients (*e.g*., nitrogen and phosphorus) with an excess of carbon [5]. To date, around 150 different types of hydroxyalkanoic acids have been found to be monomers of PHAs [6] that together with the variety of alkyl side chains give rise to an extensive family of polymers with diversified properties and potential applications [4]. Among the members of the PHAs family, polyhydroxybutyrate (PHB) is the most studied polymer [7].

Despite their renewable character and promising properties, the fraction of PHAs in the global biopolymer market is still limited by the high production costs [5]. The medium recipe for cultivation of microorganisms, the use of pure cultures, and the downstream processing contribute the most to PHAs production costs. Particularly, the medium recipe normally accounts for up to 50% of the whole process costs [8,9]. Various low-value substrates from the dairy (cheese/milk whey, dairy wastewater) [10,11], oil/paper mill (sulfite liquor, effluents) [12,13], agricultural (effluents of palm oil/soybean/fruit, spent coffee grounds oil) [11,14,15], and animal (chicken/cow manure) [16,17] sectors have been studied as carbon sources for production of PHAs; however, the requirement of pretreatment steps entails additional costs. Consequently, another substrate of cheap origin has been proposed as carbon source for PHAs production, namely volatile fatty acids (VFAs) [18]. VFAs are intermediate metabolites in anaerobic digestion (AD) that leads ultimately to biogas production. Increased attention is being given to VFAs since they can be used as platform chemicals for the production of various products more valuable than biogas and with a wider range of applications [19,20]. Therefore, attention has been given to process close control and monitoring in order to enhance VFAs production and drive away from biogas production, that is, employing an acidogenic fermentation [21]. Through microbial cultivation where the bacterial propagation is carried out under-controlled conditions of, *e.g*., temperature, pH and dissolved oxygen, VFAs are utilized as carbon sources and converted into, *e.g*., (R)-3-hydroxybutyryl-CoA and (R)-3-hydroxyvaleryl-CoA which can then be converted into polyhydroxybutyrate (PHB) and polyhydroxyvalerate (PHV), respectively [22]. Altogether, the utilization of VFAs for PHAs production will not only reduce the production costs but also have a positive impact on environmental and social aspects since VFAs can be produced through degradation of organic wastes largely available worldwide.

Besides fermentation substrates, the selection of bacterial strains for PHA production is naturally also important. Nowadays, the commercialized PHAs products are exclusively produced by Gram-negative bacteria due to its high productivity and flexibility of consuming both simple and complex substrates [2]. However, the lipopolysaccharide present in the outer membrane of Gram-negative bacteria can act as an endotoxin which hampers commercial exploitation for biomedical applications [23]. In this case, endotoxin-free Gram-positive bacteria such as *Bacillus* spp. are promising alternatives. Related advantages include high growth rate, absence of lipopolysaccharide layer which facilitates PHAs extraction, and the ability to produce hydrolytic enzymes that aid hydrolysis of more complex low-value substrates for subsequent use as carbon sources for PHAs production [24–26]. In fact, members of the genus *Bacillus* have been widely studied for PHAs production using synthetic media containing common carbon sources such as arabinose [27], glucose [27–30], glycerol [31,32], lactose [30,32], and sucrose [27,30,33]. Besides, VFAs including acetic acid [28,32], propionic acid [28], butyric acid [32], octanoic acid [32], among others, were also applied and at some extent, led to positive results. However, the effect of VFAs type on PHAs production was in focus, while further studies are needed to evaluate the effect of using mixtures of VFAs on PHAs production using a wider range of strains and substrates used for acidogenic fermentation.

The aim of the present study was to investigate the effect of VFAs on PHAs production by a strain of *Bacillus megaterium* with especial focus on VFAs type, origin, and cultivation time. VFAs were added to a synthetic medium or provided as a stream originated from acidogenic fermentation of food waste. Both bacterial strain and stream origin of VFAs have not previously been studied in the literature.

## 2. Materials and methods

### 2.1. Bacterial strain and chemicals

*Bacillus megaterium* ATCC 14,945 (American Type Culture Collection, Manassas, VA, USA) was used in this study. The bacterial culture was preserved in Lysogeny broth (LB) agar plates composed of (in g/L) agar 15, tryptone 10, NaCl 5, and yeast extract 5. New plates were prepared monthly by streaking method and followed by incubation at 28°C for two days and storage at 4–6°C until use. Glucose, synthetic VFAs (acetic acid, propionic acid, butyric acid, iso-butyric acid, valeric acid, isovaleric acid, and caproic acid) and all other chemicals for growth medium preparation and PHAs extraction were purchased from Sigma Aldrich and were all of analytical grade (≥99% purity).

### 2.2. Volatile fatty acids from acidogenic fermentation of food waste

The volatile fatty acids stream was obtained from acidogenic fermentation of food waste using a continuous 2.5 L stirred-tank bioreactor (BBI-biotech GmbH, Berlin, Germany) equipped with a membrane panel (68.6 cm^2^ effective area and average pore size of 0.3 µm) (VITO NV, Mol, Belgium). The reactor was fed with the substrate mimicking a typical food waste from European Union [34] and inoculated with bacterial seeding granules (90 g) from an upflow anaerobic sludge blanket reactor treating wastewater (Hammarby Sjöstad, Stockholm, Sweden); tap water was then added to reach a working volume of 2 L. The initial pH was set at 6 by addition of 2 M NaOH and 2 M HCl and the acidogenic fermentation was conducted at 37°C without pH control and nitrogen was sparged into the reactor for 5 min to create anaerobic conditions. The VFAs permeated through the membrane panel and were *in situ* separated by a peristaltic pump (Watson Marlow, Wilmington, UK) set at 10 rpm. The hydraulic retention time and organic loading rate were kept at 6.67 days and 2 g volatile solid/L/day, respectively. After achieving a stable stage from day 31, the organic loading rate was increased to 4 g volatile solid/L/day from day 40 to study the effect of high solids concentration on the membrane performance. The operation was conducted in semi-continuous mode by performing the filtration (210 s) and backwash (30 s) cycles by an electric relay (Zelio Logic SR2A101BD, Schneider Electric, USA) in advance to the feeding step once per day [21].

### 2.3. Substrate preparation and cultivation conditions

The minimal salt medium (MSM) used in this study was prepared with the following composition (in g/L): Na_2_HPO_4_.2H_2_O 4, KH_2_PO_4_ 3.6, (NH_4_)_2_SO_4_ 3, MgSO_4_.7H_2_O 0.5, CaCl_2_.H_2_O 0.05, NaCl 0.02, carbon source 5, trace metals, and vitamins. Glucose, acetic acid, propionic acid, butyric acid, iso-butyric acid, valeric acid, isovaleric acid, and caproic acid were prepared separately and tested as sole carbon sources; the mixture of these synthetic VFAs was also used as cultivation medium. The cultivation medium in all experiments was adjusted to pH 7 by addition of 2 M NaOH or 2 M HCl. The vitamin solution (added at 1 mL/L) contained 0.05 g/L of d-biotin, 0.2 g/L of p-aminobenzoic acid, 1 g/L of nicotinic acid, 1 g/L of Ca-pantothenate, 1 g/L of pyridoxine, 1 g/L of thiamine, and 25 g/L of m-inositol, while the trace metal solution (added at 1 mL/L) contained 3 g/L of EDTA, 0.9 g/L of CaCl_2_.H_2_O, 0.9 g/L of ZnSO_4_.7H_2_O, 0.2 g/L of H_3_BO_3_, 0.19 g/L of MnCl_2_.4H_2_O, 0.08 g/L of Na_2_MoO_4_.2H_2_O, 0.06 g/L of CoCl_2_.2H_2_O, 0.06 g/L of CuSO_4_.5H_2_O, and 0.002 g/L of KI. The VFAs-rich solution originated from acidogenic fermentation was sterilized by filtration, while all other compounds and solutions were autoclaved (Systec, Linden, Germany) at 121°C for 20 min.

Shake flasks cultivations were performed in 100 mL cotton-plugged Erlenmeyer flasks. The flasks containing 50 mL of medium were inoculated with one bacterial colony and incubated at 32°C using water baths (Keison, Chelmsford, UK) shaking at 120 rpm. The cultivations were conducted for three days and seven mL samples were taken at different times for analysis of carbon source consumption, cell growth through spectrophotometry, and pH. Total flask medium volumes were used for cell mass quantification and for PHAs extraction, quantification, and analysis following 24 h, 48 h, and 72 h of cultivation.

### 2.4. Cell dry weight measurement

The bacterial cells were harvested by centrifugation at 10,000 × g for 10 min (Megafuge 8, Thermo Fisher Scientific GmbH, Dreieich, Germany). The bacterial cells were then washed three times with distilled water followed by centrifugation and dried in an oven at 70°C (TERMAKS, Bergen, Norway) to constant weight. The cell dry weight (CDW) is either reported as grams of cells per liter of medium or as grams of cells per gram of consumed carbon source.

### 2.5. PHA extraction

The extraction of PHAs was performed according to Hahn, et al. [35] with modification. Briefly, the bacterial cells were firstly collected by centrifugation at 10,000 × g for 10 min followed by three times rinsing with distilled water and incubation in sodium hypochlorite (10–15%) at 30°C for 1 h. Afterward, the PHAs were separated from the solution by centrifugation at 10,000 × g for 10 min and rinsed with distilled water, acetone, and absolute ethanol to remove remaining cell debris. The centrifugation was applied in each washing step and then hot chloroform was added to dissolve the PHAs followed by filtration to remove non-PHAs material. Finally, a solution of methanol and water (7:3 v/v) was used to precipitate the PHAs. The obtained PHAs was placed in a pre-weighted aluminum cup and dried in an oven at 70°C (TERMAKS, Bergen, Norway) to constant weight. The percentage of PHAs based on cell dry weight was quantified by the following equation:

$$PHAs\left(\%\right)=\frac{PHAsweight}{Celldryweight}\times 100$$

Based on this equation, the concentration of PHAs per liter of solution was also calculated and reported.

### 2.6. Determination of carbon source consumption and cell growth

The determination of carbon source consumption was performed by high-performance liquid chromatograph (HPLC) (Waters Corporation, Milford, CT, USA) with a hydrogen-based column (Aminex HPX87, BioRAD Laboratories, Munich, Germany), H_2_SO_4_ (5 mM) as eluent at a rate of 0.6 mL/min, and a refractive index detector (Waters Corporation, Milford, CT, USA). Regarding the determination of VFAs consumption, an ultraviolet (UV) absorption detector operating at 210 nm wavelength (Waters 2487, Waters Corporation, Milford, CT, USA) was used in series with the refractive index detector. All samples were kept in 2 ml Eppendorf tubes and centrifuged (Fresco 21, Thermo Lectron LED GmbH, Osterode, Germany) at 10,000 × g for 10 min. The supernatants were then filtered using 0.2 µm pore size filters before being transferred into vials for HPLC analysis.

For the cell growth monitoring, a sample of one mL was collected from the cultivation flasks and analyzed in a spectrophotometer at a wavelength of 600 nm for optical density (OD) measurement.

### 2.7. PHA characterization

#### 2.7.1. Fourier-transform infrared spectroscopy (FTIR)

FTIR was used for analysis of functional groups in the sample structure. The analysis was conducted by directly scanning the sample 64 times in a spectrum of 400 to 4000 cm^−1^ by Nicolet OMNIC 4.1 software. The obtained spectra were then analyzed by Essential FTIR (eFTIR, Madison, WI, USA).

#### 2.7.2. Differential scanning calorimetry (DSC)

The thermal properties of the extracted sample were determined by differential scanning calorimeter (Q500 TA instruments, Waters LLC, New Castle, DE, USA). Three milligrams of each sample were heated from −40°C to 225°C at a rate of 10°C/min. The measurement was conducted in triplicate under nitrogen atmosphere. The values were taken from the second heating and the crystallinity was calculated by the following equation:

$$\Delta X=\frac{\Delta H}{\Delta H_{m}^{o}}$$

where ΔΗ is the specific enthalpy of fusion (in J/g) of the extracted sample determined from the peak area and ΔΗ°_m_ (146 J/g) is the enthalpy of fusion of a 100% crystalline PHB.

### 2.8. Analysis and presentation of results

All experiments were carried out in duplicate and the same number of analytical analyses were performed with the exception of DSC tests that were carried out in triplicate per sample. The results obtained are presented in the form of average values ± 2 times the standard deviation in both tables and graphs.

## 3. Results and discussion

Volatile fatty acids (VFAs) are intermediate products in the anaerobic digestion leading to the sequential breakdown of organic wastes through four fundamental biochemical reactions, namely hydrolysis, acidogenesis, acetogenesis, and methanogenesis [36]. Organic wastes comprising carbohydrates, proteins, and lipids are hydrolyzed into their corresponding monomers, *i.e*., sugars, amino acids, and fatty acids. These monomers function as substrates in the acidogenesis step where they are broken down into VFAs, alcohols, lactate, CO_2_ and H_2_ [37]. The acidogenic products are further degrade into acetic acid, CO_2_ and H_2_, during acetogenesis, which are finally converted into CH_4_ by methanogenic archaea [37]. However, VFAs have more recently been considered as platform chemicals for various applications, *e.g*., production of polyhydroxyalkanoates, and hence of higher potential value than that of biogas [19]. Therefore, there is increasing research interest on the inhibition of methanogenesis to enhance VFAs accumulation, that is, carrying out an acidogenic fermentation. An example of a group of reactions converting sugars to acetic acid during anaerobic digestion is presented below. The last two reactions are inhibited to prevent CH_4_ production and enhance VFAs accumulation.

$$\begin{array}{c} {(C_{6}H_{10}O_{5})}_{n}+H_{2}0\to_{n}C_{6}H_{12}O_{6}\\ C_{6}H_{12}O_{6}+2H_{2}\to 2CH_{3}CH_{2}COOH+2H_{2}O\\ CH_{3}CH_{2}COOH+3H_{2}O\to CH_{3}COOH+H_{2}O+CO_{2}\\ CH_{3}COOH\times \to CH_{4}+CO_{2}\\ CO_{2}+4H_{2}\times \to CH_{4}+2H_{2}O \end{array}$$

**Figure 1.**
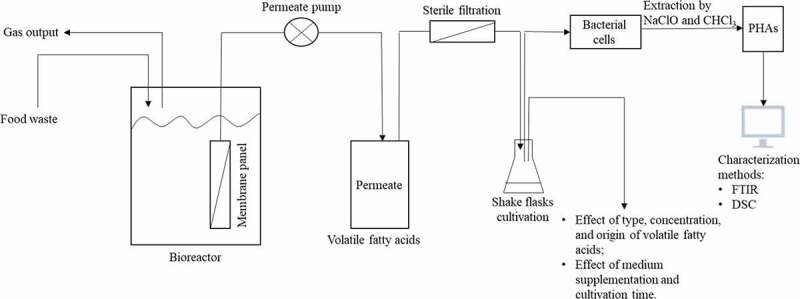
Scheme showing the integration of VFAs production through acidogenic fermentation of food waste using membrane bioreactors with PHAs production by Bacillus megaterium ATCC 14,945 through shake flask experiments together with factors studied and analyses carried out in this work

The VFAs-rich stream used in this work was obtained from the acidogenic fermentation of food waste which was conducted in an immersed membrane bioreactor. With the assistance of membranes, VFAs can be easily and continuously recovered for further processing (Figure 1) [21]. VFAs can be used for PHAs production; therefore, the aim of this work is to directly utilize the VFAs from acidogenic fermentation of food waste as a carbon source, whereby, reducing the high cost of commercial PHAs production. In a first stage of the present work, a defined medium containing single or a mixture of synthetic volatile fatty acids, purchased from common suppliers, were used for growth of *Bacillus megaterium* ATCC 14,945 in order to evaluate potential inhibitory effects and substrate preference; glucose was used as reference carbon source. The obtained results were then compared with those obtained through bacterial cultivation in the VFAs-rich stream obtained from acidogenic fermentation of food waste (Figure 1). The ability of using VFAs as a cultivation medium would not only provide a cheap substrate for PHA production but also lighten the waste management hurdle by utilizing food waste.

### 3.1. Bacillus *sp. cultivation on glucose – and VFAs-based synthetic medium*

**Figure 2.**
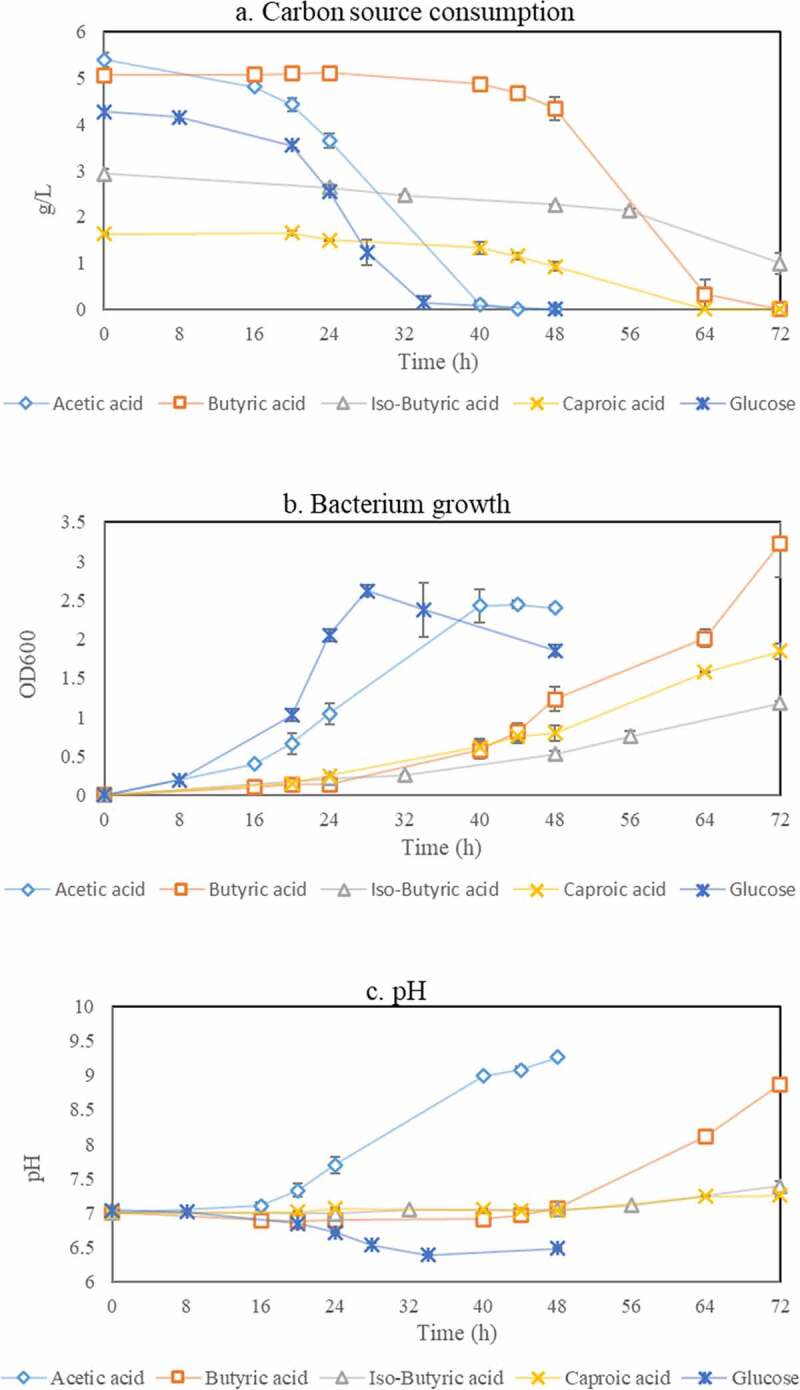
Profile of carbon source concentration (a), absorbance (b), and pH (c) during cultivation of *Bacillus megaterium* ATCC 14,945 in medium containing glucose, acetic acid, butyric acid, iso-butyric acid, and caproic acid as single carbon sources

*Bacillus* spp. are well-known to being able to accumulate PHAs from sugars [27,30,32]. However, their ability to assimilate volatile fatty acids is still scarcely described in the scientific literature. Therefore, in this work, commercially available synthetic VFAs, namely acetic acid, propionic acid, butyric acid, iso-butyric acid, valeric acid, iso-valeric acid, and caproic acid were examined, at a first stage, as single carbon sources for growth of a strain of *Bacillus megaterium*, where glucose was used as reference. The profiles of carbon source concentration, absorption, and pH during bacterial cultivation are presented in Figure 2.

It can be observed that there were differences in the consumption trend of each carbon source (Figure 2(a)). Briefly, glucose and acetic acid were found to be preferable substrates for *Bacillus megaterium* and were followed by butyric acid, iso-butyric, and caproic acid. This can be explained according to the intermediates produced during the metabolic conversion of these components. In normal condition, acetic acid is directly converted into acetyl-CoA while glucose is first converted into pyruvate which is further converted into acetyl-CoA that afterward enters the Krebs cycle to produce adenosine triphosphate (ATP) providing energy for bacterial growth [38,39]. On the other hand, the coenzyme A derivatives of butyric acid, caproic acid, propionic acid and valeric acid are butyryl-CoA, propionyl-CoA, hexanoyl-CoA and valeryl-CoA, respectively [40]. Among these components, butyryl-CoA, iso-butyryl-CoA and hexanoyl-CoA afterward can be converted into acetyl-CoA while the rest of derivatives do not participate in the Krebs cycle [41], therefore, they are assumed to be undesirable in normal bacterial growth. Furthermore, when nutrients other than carbon source are limited, the acetyl-CoA is inhibited from entering the Krebs cycle [42,43]. Consequently, the excess acetyl-CoA together with other coenzyme derivatives are directed to PHAs synthesis [38,41,44]. Some fundamental reactions are presented below:

C_6_H_12_O_6_ (glucose) → 2 C_3_H_4_O_3_ (pyruvate) + 4 H^+^ + 4e^−^

C_3_H_4_O_3_ (pyruvate) + HS-CoA (coenzyme A) → C_2_H_3_OS-CoA (acetyl-CoA) + CO_2_

CH_3_COOH (acetic acid) + CoA-HS → CH_3_COS-CoA (acetyl-CoA) + H_2_O

2CH_3_COS-CoA (acetyl-CoA) – CoA-HS → C_4_H_5_O_2_S-CoA (acetoacetyl-CoA)

C_4_H_5_O_2_S-CoA (acetoacetyl-CoA) + NaDH → C_4_H_6_O_2_S-CoA (3-hydroxybutyrul-CoA) + NAD^+^

nC_4_H_6_O_2_S-CoA (3-hydroxybutyrul-CoA) – CoA-HS → n(C_4_H_6_O_2_) (polyhydroxybutyrate)

Moreover, the initial concentrations of iso-butyric acid and caproic acid needed to be lower (2.5 g/L and 1.5 g/L, respectively) than those of acetic acid and glucose to achieve consumption, although longer times were still needed for partial or total consumption. The bacterium was not able to grow when valeric acid, iso-valeric, and propionic acid were present in the medium independently of the concentration. The results obtained are in accordance with previous studies stating the faster assimilation of carbon sources such as glucose and acetic acid compared to that of longer and branched volatile fatty acids [40]. Furthermore, it should be noted that the rate of carbon assimilation can highly depend on the environmental factors of temperature, pH and oxygen demand which directly affect bacterial activity [45–47]. For example, in this case *Bacillus megaterium* is a mesophilic bacterium, which grows in an optimal temperature of 30–37°C [48]. Above or below this range of temperature, the structure of the bacterium is less stable and the flexibility of its protein synthesis will be reduced [49,50]. The proteins synthesized in response to the needed hydrolysis and assimilation of carbon source are denatured by changes of pH [51,52]. Generally, the high number of bacterial cells will lead to the enhancement of the carbon assimilation rate. However, when it reaches a certain level of cells, the oxygen demand will increase requiring higher shaking speed during cultivation [53,54]. Therefore, the determination of optimal conditions for the cultivation step is an essential factor to increase bacterial cells productivity.

Naturally, the consumption trends of carbon sources observed were translated into absorbance profiles which cell cycles resemble the difficulty level of *Bacillus megaterium* to consume the different carbon sources used (Figure 2(b)). The pH profiles (Figure 2(c) which show an overall increasing trend during cultivation, except that for growth in glucose as single carbon source, are also in accordance with the consumption of acids and therefore, pH control should be considered in future experiments employing cultivation at reactor scale.

### 3.2. Bacillus *sp. cultivation in a mixture of VFAs derived from acidogenic fermentation of food waste*

Studies on VFAs utilization for the production of PHAs by *Bacillus* spp. is scarce in literature and are mostly limited to the single use of individual synthetic VFAs [28,32]. In this study, a mixture of VFAs derived from acidogenic fermentation of food waste was applied for the cultivation of *Bacillus megaterium*. The composition of the VFAs-rich stream is presented in Table 1. It can be observed that acetic acid, butyric acid, and caproic acid are the dominant metabolites in the stream and the remaining volatile fatty acids accounted for a very small share of the total VFAs concentration of 5 g/L. From the results presented in previous section, this VFAs-rich stream is promising for the cultivation of *Bacillus megaterium* as the bacterium can assimilate the three dominant compounds, namely acetic acid, caproic acid, and butyric acid, at the provided concentrations.

**Table 1.** Characterization of the VFAs-rich stream derived from acidogenic fermentation of food waste that was used in this study

Composition	Value (g/L)
Acetic acid	2.1–2.4
Caproic acid	1.8–2.0
Butyric acid	1.2–1.4
Iso-butyric acid	0.08–0.1
Valeric acid	0.2–0.3
Iso-valeric acid	0.1–0.2
Propionic acid	0.5–0.6

**Figure 3.**
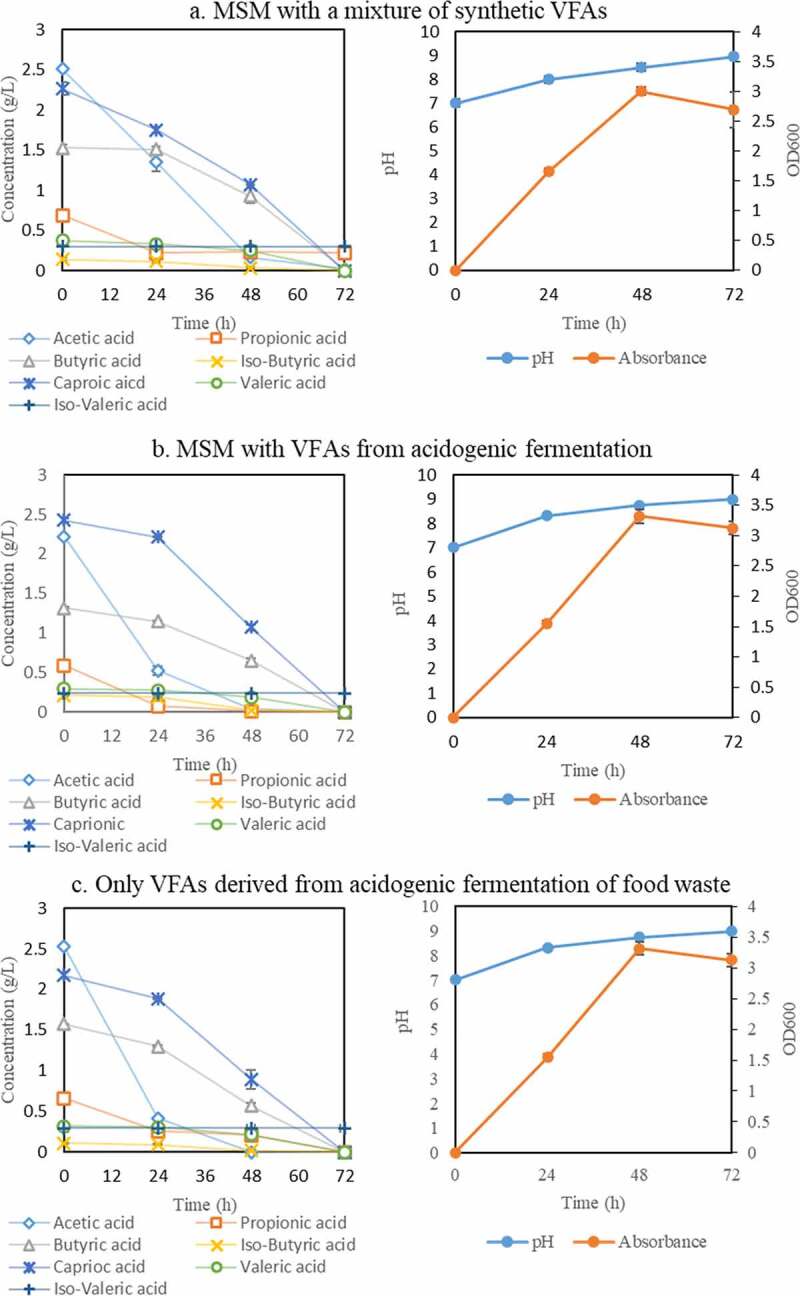
Profiles of VFAs concentration, absorbance, and pH during cultivation of *Bacillus megaterium* ATCC 14,945 in three different cultivation media: a) Minimum Salt Medium (MSM) with a mixture of synthetic VFAs; b) MSM with VFAs from acidogenic fermentation; and c) VFAs-rich stream as produced from acidogenic fermentation of food waste

In this assay, the effect of three different cultivation media, including minimal salt medium (MSM) with a mixture of commercially available synthetic VFAs (medium A), MSM with VFAs from acidogenic fermentation (medium B), and the VFAs-rich stream from acidogenic fermentation as obtained from the membrane bioreactor (medium C), on carbon source assimilation by *Bacillus megaterium* was studied. The composition of the synthetic VFAs mixture was prepared according to the medium obtained from the acidogenic fermentation with a total concentration of around 7 g/L. The profiles of carbon source consumption, absorbance, and pH during bacterial cultivation are presented in Figure 3.

The VFAs were almost depleted in all three media after 72 h of cultivation. The total reduction of the initial VFAs concentrations in the medium A, B, and C were 96.1, 97.1, and 96.1%, respectively. The trends of VFAs consumption from the bacterial growth in the three media were similar with the exception of the consumption rate of acetic acid. After 24 h, the acetic acid reduction by the bacterium in medium A was 48% which was relatively lower than that in medium B (77%) and C (84%). After 48 h of cultivation, this component was completely depleted in medium B and C while 0.2 g/L remained in medium A. Additionally, the VFAs, namely iso-butyric acid and propionic acid, which were not individually consumed by the bacterium were now assimilated in all medium recipes. A potential explanation for the obtained results can be that the bacterial growth in more easily consumed VFAs enabled the consumption of the remaining VFAs at later stages of the cultivation by tackling their potential inhibitory effects. This mechanism can be elucidated by the oxidation process of the volatile fatty acids producing different coenzyme A derivatives of acetyl-CoA, butyryl-CoA, isobutyryl-CoA, hexanoyl-CoA, propionyl-CoA, valeryl-CoA and isovaleryl-CoA as intermediates [55]. The initial energy that is used to oxidize these components can be derived from the utilization of other nutrients in the MSM. Moreover, it was proven that only acetyl-CoA and derivatives, namely butyryl-CoA, isobutyryl-CoA, hexanoyl-CoA can be converted into acetyl-CoA further supporting the bacterial growth [14,40,56]. This is in accordance with the obtained result in Section 3.1 where *B. megaterium* could grow on acetic acid, butyric acid, isobutyric acid and caproic acid as single carbon source. On the other hand, the bacterium might be able to oxidize propionic acid, valeric acid and iso-valeric; however, their derivatives could not enter the Krebs cycle to produce the energy leading to growth inhibition. Consequently, during cultivation in a mixture of VFAs, the bacterium can firstly grow on acetic acid, butyric acid and caproic acid obtaining energy to continuously oxidize propionic acid and iso-valeric acid. The non-consumption of valeric acid is assumed to be related to the lack of enzymes being able to oxidize this component.

The obtained results are promising as they revealed that the bacterial growth can occur in the VFAs-rich stream from acidogenic fermentation without any nutrient supplementation. Therefore, the VFAs-rich stream is hypothesized to contain nutrients supporting the growth of the bacterium which needs further analytical investigations.

### 3.3. *Cell dry weight and PHA accumulation following* Bacillus *sp. cultivation in glucose- and synthetic VFAs-containing medium*

**Figure 4.**
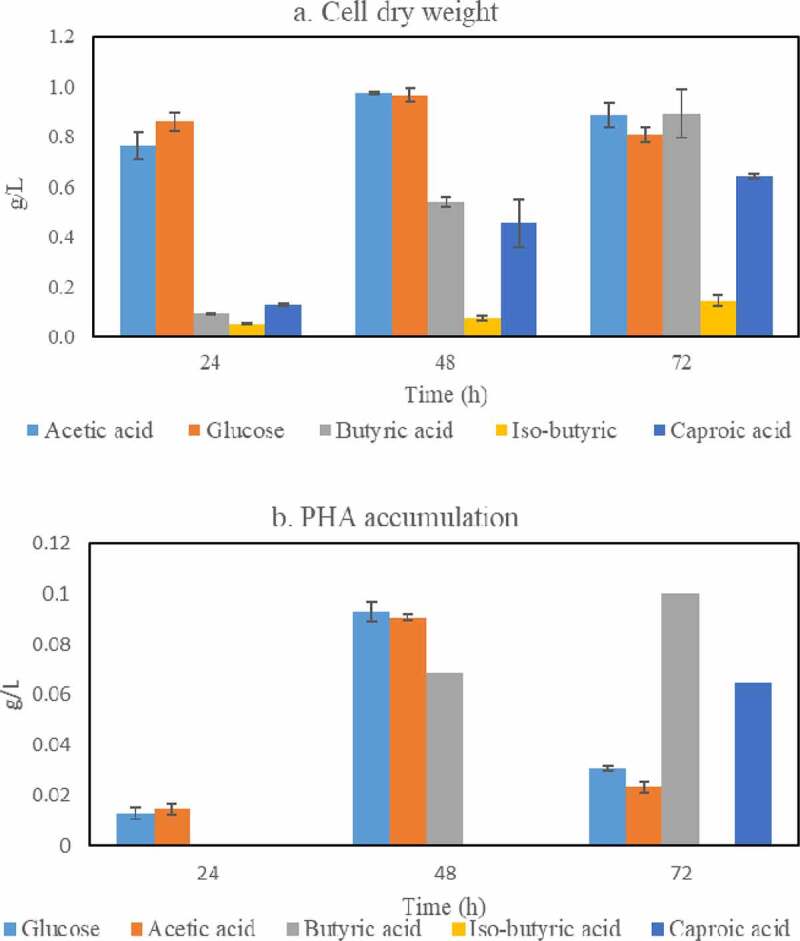
Cell mass (a) and PHAs (b) concentrations obtained during cultivation of *Bacillus megaterium* in medium containing glucose, acetic acid, butyric acid, iso-butyric acid, and caproic acid as single carbon sources

Based on the results reported in Section 3.1, five carbon sources, namely glucose, acetic, butyric, iso-butyric, and caproic acids were used to assess the ability of *Bacillus megaterium* ATCC 14,945 to accumulate PHAs. In this experiment, the cultivation was carried out for three days with sampling following 24 h, 48 h, and 72 h of cultivation for the measurement of cell dry weight and PHA accumulation; and the results are presented in Figure 4.

In general, the patterns of cell dry weight and PHAs production using glucose and acetic acid as carbon sources are similar, while butyric acid, iso-butyric acid, and caproic acid only share similar cell dry weights. Specifically, the maximum cell dry weight and PHAs accumulation were reached after 48 h of cultivation using glucose and acetic acid as single-carbon sources: cell dry weight of 0.96 and 0.97 g/L (0.22 and 0.19 g/g carbon source), and PHAs accumulations of 0.093 and 0.091 g/L (0.096 and 0.093 g/g CDW) were obtained in glucose- and acetic acid-containing medium, respectively. After this time, the cell dry weight slightly decreased while a significant reduction in PHAs accumulation was observed. This can be explained by the characteristic consumption of PHAs as carbon and energy reserves to lengthen the survival of the bacterium after the depletion of carbon sources [57]. On the other hand, the cell dry weight originated from cultivation in butyric-, iso-butyric- and caproic acid-containing medium as single carbon sources increased over time reaching their peaks after 72 h, namely of 0.89, 0.15, and 0.64 g/L (0.18, 0.07 and 0.39 g/g carbon source), respectively. However, PHAs accumulation only occurred during cultivation in butyric and caproic acid-containing medium as single carbon sources where the highest amounts of 0.1 and 0.06 g/L (0.11 and 0.09 g/g CDW) were also recorded after 72 h of cultivation. There was no PHAs accumulation during cultivation in iso-butyric acid-containing medium which is hypothesized to be related to the lack of PHAs synthase that can utilize the coenzyme A derivative from iso-butyric.

Compared to the bacterial cultivation in glucose and acetic acid, the slower production of PHAs in butyric acid and caproic acid can be explained by their PHAs synthesis pathways. Instead of being converted directly to acetyl-CoA for PHAs production, butyric and caproic acid have to go through several reactions before being channeled into PHAs biosynthesis. In fact, butyric acid is converted into butyryl-CoA which can be further converted into acetyl-CoA or 2-butanoyl-CoA [40]. The latter will continue to be converted into R-3-hydroxybutyryl-CoA and acetoacetyl-CoA for PHAs production. Caproic acid, otherwise, is converted into caproyl-CoA which undergoes β-oxidation and transformed back to acetyl-CoA due to the inability of *B. megaterium* to produce medium chain length PHAs [26,42,58]. Those reactions are assumed to require more extra energy and cause delays in PHAs production during cultivation in butyric acid and caproic acid as single carbon source.

Altogether, the obtained PHAs yields, based on cell dry weight, were within the range of 9–12% which is similar to that reported in previous studies using other *Bacillus* spp. (Table 2) [23,40,58]. Additionally, the present study showed, for the first time, the ability of a *Bacillus* sp. to produce PHAs from butyric and caproic acid as single carbon sources.

**Table 2.** Comparison of PHAs yields on a cell mass weight obtained in this work and in previous published studies

**Microorganisms**	**Carbon substrate and concentration (g/L)**	**PHA type & yields (%)**	**Cultivation type**	**Reference**
*Bacillus megaterium ATCC14945*	Glucose, 5 g/L	PHB, 10%	Batch	This work
	Acetic acid, 5 g/L	PHB, 9%		
	Butyric acid, 5 g/L	PHB, 11.2%		
	Caproic acid, 1.8–2 g/L	PHB, 9.2%		
	a mixture of synthetic VFAs	PHB, 10%		
	a mixture of VFAs from AD, 7 g/L	PHB, 8.6%		
*Bacillus cereus*	Glucose, 5 g/L	PHB, 13.7%	Batch	[75]
*Bacillus thuringiensis*	Glucose, 5 g/L	PHB, 11.3%	Batch	[76]
*Bacillus cereus* SPV	Glucose, 20 g/L	PHB & PHV, 38%	Batch	[61]
*Bacillus megaterium* DSM 90	Propionic acid, 1.5 g/L			
	PHBV, 33.7%	Batch	[77]	
*Ralstonia eutropha*	Acetic acid, 5 g/L	PHB, 29.3%	Batch	[78]
	Butyric acid, 5 g/L	PHB, 31.9%		
*Cupriavidus necator*	Acetic acid, 15 g/L	PHB, 34%	Fed-batch	[79]
	Butyric acid, 25 g/L	PHB, 19%		

### 3.4. *Cell dry weight and PHA accumulation by* Bacillus *sp. during cultivation in VFAs-rich stream derived from acidogenic fermentation of food waste*

**Figure 5.**
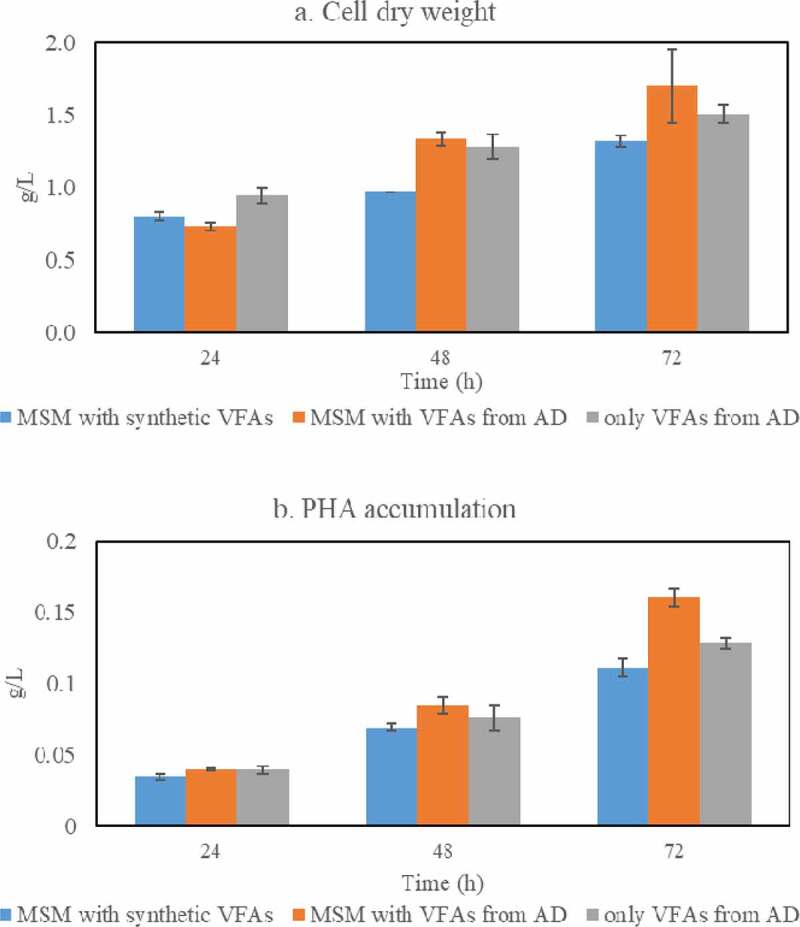
Cell mass (a) and PHAs (b) concentrations obtained during cultivation of *Bacillus megaterium* ATCC 14,945 in three different medium recipes: a) Minimal Salt Medium (MSM) containing a mixture of synthetic VFAs; b) MSM with VFAs from acidogenic fermentation; and c) VFAs-rich stream as produced from acidogenic fermentation of food waste

The cell dry weight and PHAs accumulation were also monitored during cultivation of *Bacillus* sp. in the three media reported in Section 3.2.; the findings are presented in Figure 5. The cell dry weights and PHAs accumulations were similar among the media studied and reached their maximum after 72 h of cultivation; cell dry weight of up to 1.70 g/L (0.23 g/g carbon source) and PHAs accumulation of up to 0.16 g/L (0.09 g/g CDW) were obtained. Altogether, PHAs accumulation of 8–9%, on a cell dry weight basis, was achieved which was similar to that obtained when glucose or different volatile fatty acids were used as single carbon sources during cultivation of *Bacillus* sp.

### 3.5. PHA characterization

#### 3.5.1. Fourier-Transform Infrared Spectroscopy (FTIR) analysis

**Figure 6.**
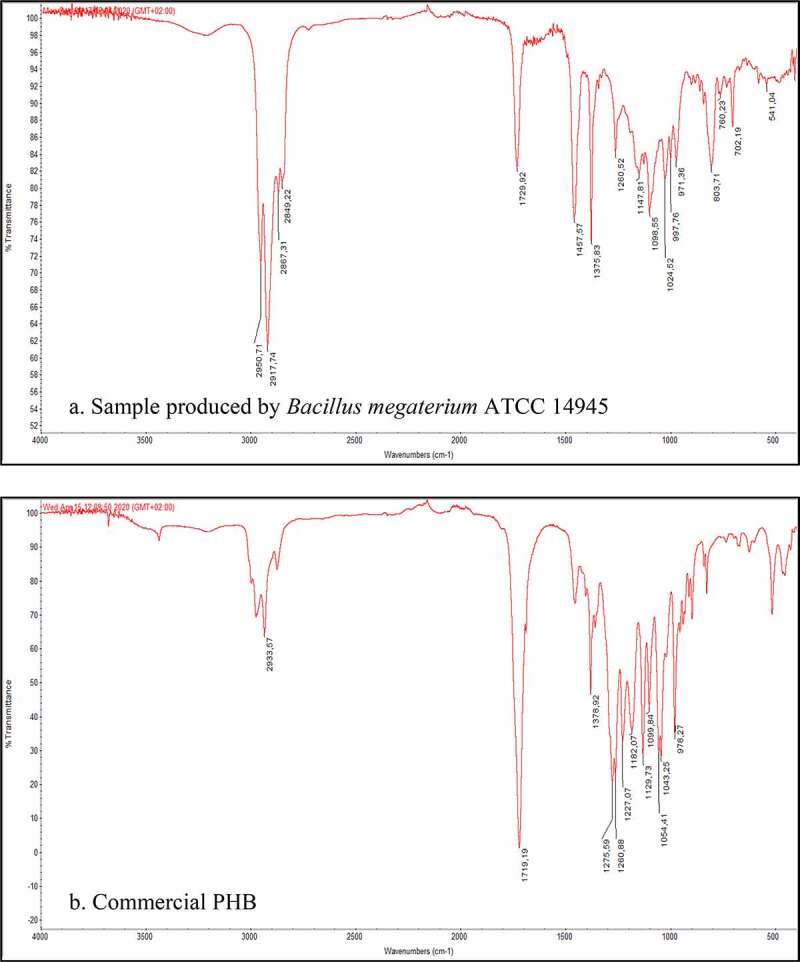
Comparison of FTIR spectra obtained from the analysis of PHAs extracted from bacterial cell grown in VFAs-rich stream from acidogenic fermentation of food waste (a) and of a commercial PHB sample (b)

The FTIR spectrum of the extracted sample of PHA from bacterial cell grown in food waste acidogenic fermentation-derived VFAs-rich stream is presented in Figure 6(a). The FTIR analysis revealed the intense peaks at 1735 and 1260 cm^−1^ indicating the typical ester carbonyl group of PHB which is similar to and confirmed by previous studies employing *Bacillus* spp [59–61]. Other common stretches of CH_3_, CH_2_, and CH groups were observed by the peaks at 1377, 1452, and 1222 cm^−1^, respectively, which are also representative chemical structures of PHB [2,62]. The obtained spectrum is also compared to that of the commercial PHB in Figure 6(b).

#### 3.5.2. Differential Scanning Calorimetry (DSC) analysis

**Figure 7.**
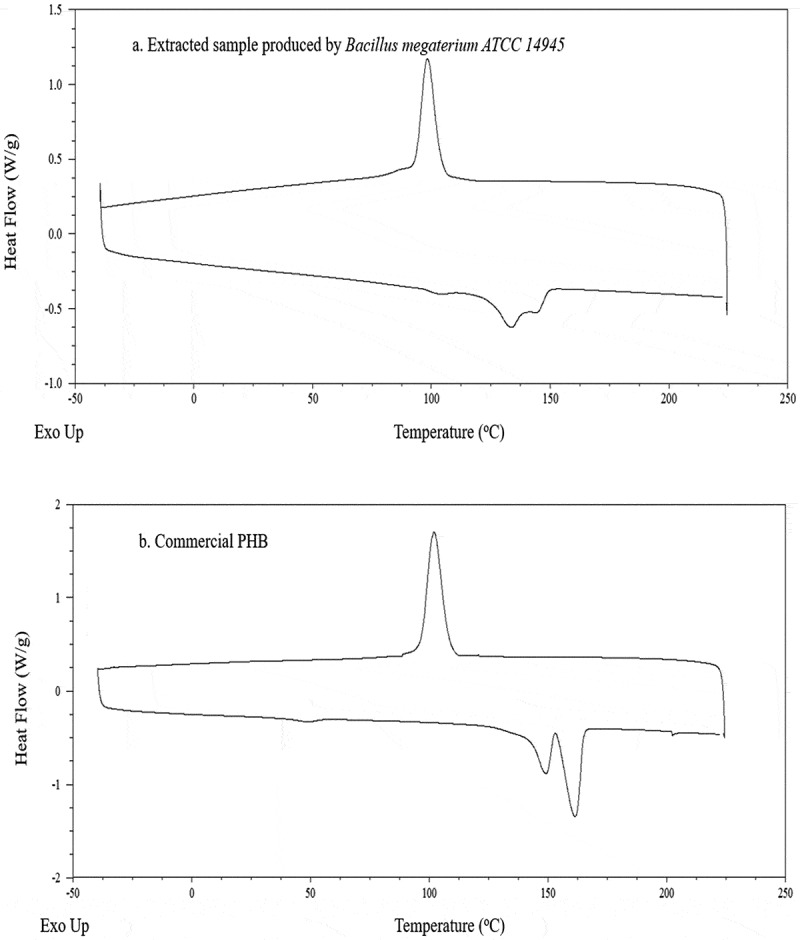
Comparison of DSC profiles between PHAs extracted from bacterial cell grown in VFAs- rich stream from acidogenic fermentation of food waste (a) and a commercial PHB sample (b)

The melting temperature (T_m_) of the extracted sample was analyzed by DSC and the result is presented in Figure 7(a) and Table 3. The endothermic peaks of the extracted sample were obtained at 133.15 and 145.42°C which were lower than those obtained for the commercial PHB (148.89 and 161.62°C). The obtained results were found similar to those obtained for the extracted PHB from *Bacillus subtilis* which had two melting temperatures at 120 and 145°C [63]. Another low melting temperature for PHA recovered from recombinant *Escherichia coli* cells originated from cultivation in molasses has been reported [64]. Regardless the difference in melting temperature, the extracted sample and the commercial PHB showed the same crystallization temperature at around 100°C. From a commercial perspective, melting and crystallization temperatures are important factors that define the product quality and its final applications [65–67]. In fact, plastics that are used in general purpose, engineering and high temperature specialty have melting temperatures of around 100, 150 and 300°C, respectively [68,69]. Besides, at crystallization temperature the molecules are mobile enough to allow rearrangement into ordered structures that affect storage conditions and avoid brittleness [70]. Therefore, the extracted PHB can be a potential option for general applications to replace the use of conventional plastics which do not require exposure to high temperature.

**Table 3.** DSC results of PHAs extracted from bacterial cell grown in VFAs-rich stream originated from acidogenic fermentation of food waste and that of a commercial PHB sample

Sample	T_m_ (^o^C)	ΔH (J/g)	X_c_ (%)
Extracted PHAs	133.15–145.42	18.71	12.81
Commercial PHB	148.89–161-62	74.09	50.74

Moreover, the crystallinity degrees (X_c_) of the extracted PHB and of the commercial PHB were 12.81 and 50.74%, respectively. They were measured based on the melting enthalpy (ΔH) of the endothermal peaks of 18.71 and 74.09 J/g, respectively. Additionally, the presentation of dual melting point in the extracted sample can be explained by the reactions of re-crystallization and cross-linking isomerization which demonstrates the nature of PHB as the commercial PHB also presented the same phenomenon [71].

## 4. Practical applications and future research perspectives of this work

Nowadays, PHB is commercially produced by fermentation process of pure bacterial strains under optimal conditions using mostly refined sugars. Some PHAs manufacturers namely Biomer, Bio-on, Tepha, Tianan Biopolymer, etc. provide PHAs prices of 2–10 US$/kg which are still 6–10 times higher than those of the traditional plastics [72–74]. In addition to the cost of fermentation medium recipe, the utilization of pure cultures requiring sterile conditions as well as the complexity of the extraction and recovery of PHB are important factors to take into account while aiming at establishing feasible processes for PHB production.

In this work, a new strain of *Bacillus megaterium* was studied for PHAs production and the quality of the extracted PHB was found to be suitable for packaging applications. At a first glance, several factors such as substrate concentration, cell dry weight, and PHB yield need to be improved in future research studies. The continuous research on optimization of the use of immersed membrane bioreactors systems for acidogenic fermentation will undoubtedly lead to increased VFAs concentrations. Coupling also the bacterial fermentation process to membrane bioreactors can help achieving higher bacterial cell densities and consequently increase cell and PHB productivities. Therefore, a whole set of research initiatives toward strain screening and microbial growth optimization are needed at higher concentrations of VFAs. In addition to reactor-scale studies, techno-economic analyses need to start to come to light in order to reveal hotspots that need to be in focus in order to obtain feasible VFAs-based processes for PHB or others PHAs production.

The present work provides a first characterization of the process of PHAs production from VFAs, generated from acidogenic fermentation of a novel low-value substrate, namely food waste. Besides food waste, the sources of raw materials for acidogenic fermentation are diverse including animal manure, agricultural waste, municipal waste, etc., and therefore continuous screening of wastes for VFAs production should take place to reveal the most promising substrates. A great deal of research, development, and commercial exploitation of these substrates, in the form of anaerobic digestion processes has taken place all over the world. Anaerobic digestion is well-established worldwide as one of the most contributing technologies to waste management. Therefore, potential exists for worldwide conversion of anaerobic digestion plants into VFAs-producing plants that can feed into PHAs-producing plants ultimately contributing to the commercial expansion of the latter. The utilization of anaerobic digestion facilities for the scale up of PHAs production is believed to be beneficial where most of the equipment is available lowering initial investment costs together with a more diversified range of applications for PHAs (and VFAs) in comparison to those assigned to biogas, namely as transport biofuel, or for heat and electricity generation.

Furthermore, PHAs are biodegradable; therefore, the PHAs-based waste materials can readily be recycled through acidogenic fermentation and reconverted into VFAs. This will, in turn, open up the possibility of developing closed-loop processes for PHAs production and recycling and mitigate environmental pollution. Altogether, there is a high potential for PHAs production from acidogenic fermentation-derived VFAs; however, systematic studies addressing a range of key elements such as waste sorting at source, optimization of acidogenic fermentation, optimization of bacterial fermentation toward PHAs production, alignment between PHAs extraction and purification methods and final applications, as well as techno-economic analyses and life-cycle assessments need to take place in the future.

## 5. Conclusions

In this study, volatile fatty acids from acidogenic fermentation of food waste proved to be a potential substrate for the production of PHAs by *Bacillus megaterium* ATCC 14,945, which tackle the need of medium supplementation. The type of VFAs presented in the stream originated from acidogenic fermentation and the bacterial strain were found to be a suitable fit since all VFAs, excluding valeric acid, could be consumed and PHAs were accumulated. The PHB yields, per dry cell weight basis, as well as the final dry cell weights obtained in this work call for the need of further studies on strain screening and/or growth optimization in order to achieve higher cell densities, higher PHAs production yields and productivities. It is hypothesized that the cultivation output such as productivity due to high cell density can be improved by transferring the knowledge obtained from coupling immersed membrane bioreactors to acidogenic fermentation into the bacterial fermentation stage toward PHAs production.

## Highlights

- Volatile fatty acids (VFAs) are cheaper substrates for biopolymer production
- Unreported polyhydroxyalkanoate production using butyric and caproic acids
- Using food waste acidogenic fermentation-derived VFAs bypassed nutrient addition
- VFAs-grown *Bacillus megaterium* dry cells contained up to 9% polyhydroxybutyrate
